# Metal Levels in Southern Right Whales (*Eubalaena australis*) from Península Valdés, Argentina

**DOI:** 10.4172/2161-0525.1000190

**Published:** 2013-09-18

**Authors:** Julieta Martino, Sandra S Wise, Christopher Perkins, Mariano Sironi, John Pierce Wise

**Affiliations:** 1 Wise Laboratory of Environmental and Genetic Toxicology, Maine Center for Toxicology and Environmental Health, Department of Applied Medical Sciences, University of Southern Maine, 96 Falmouth St., Portland, ME 04104, USA; 2 Instituto de Conservación de Ballenas, CC 39-1623, Buenos Aires, Argentina; 3 Center for Environmental Sciences and Engineering, University of Connecticut, 3107 Horsebarn Hill Road, Building 4 Annex, U-4210 Storrs, CT 06269-4210, USA; 4 Cátedra de Diversidad Animal II, Facultad de Ciencias Exactas, Físicas y Naturales, Universidad Nacional de Córdoba, Av. Vélez Sársfield 299, 5000, Córdoba, Argentina

**Keywords:** *Eubalaena australis*, Southern right whale, Península Valdés, Metals, Chromium, Aluminum, Barium

## Abstract

Península Valdes, Argentina, is a nursing ground for a population of southern right whales (*Eubalaena australis*). In the last two decades this area has been subjected to an increase in population, tourism and industrial growth. This has raised the concern for exposure to chemical contaminants such as metals. In this study we measured nonessential metals (Ag, Al, As, Au, Ba, Be, Co, Cd, Cr, Hg, Li, Ni, Pb, Sb, Sn, Sr, U and Ti), essential metals (Cu, Fe, Mg, Mn, Mo and Zn) and an essential element (Se) in skin biopsies from female southern right whales (n=10). This is the first report on tissue concentrations of metals in adult southern right whales. Overall, tissue values were on the low end of the spectrum and similar to the values reported in studies of mysticetes from other regions. Measured values do not reflect substantial amounts of accumulation and thus serve as a baseline.

## Introduction

The waters around Península Valdes (PV), Argentina, are a nursing ground for a population of southern right whales (*Eubalaena australis*) [1,2]. In the last two decades, and following the trends of other coastal areas of the world, this area has been subjected to an increase in population, tourism and industrial growth. This scenario raises the concern for an increase of contaminants related to these activities and their potential effects on wildlife.

Among contaminants, metals are of particular interest because they are elements and, therefore, cannot be degraded. Thus, they can persist in the environment and be incorporated into organisms, through dermal absorption, inhalation or ingestion, where they accumulate until excreted [3,4]. Metals affect multiple cellular and organ functions, including reproduction and development [5,6]. Even essential metals can have deleterious health effects if their concentrations are too high. Specifically, in cetaceans, metal exposure has been correlated with infectious disease mortality, parasitic infections and pneumonias and histopathological changes in lung and kidney tissues [7–9]. Cetacean cell culture studies showed that metals can induce cytotoxic, immunotoxic and genotoxic effects [10–17]. Thus, animal and tissue culture studies suggest that metals could be a health concern to cetaceans.

At Península Valdés, Argentina sources of metals include mining, storage and transport of petroleum, harbor activities and cities that have settled in the area and are expanding [18]. The only aluminum smelter in the country (ATUAR) is located in the city of Puerto Madryn on Golfo Nuevo, the southern gulf of the Peninsula. In addition, sea currents moving in north-south direction bring waters from the Buenos Aires coast which is the most populated and industrialized area of the country with numerous metallurgical, petrochemical, textile and pharmaceutical industries [19]. This suggests that exposure to metals from contaminated water and air is possible for southern right whales that use the PV nursery ground annually from June to December. Moreover, in the last seven years this population has been experiencing high calf mortalities [20–22] and environmental contaminants have been suggested as a contributing factor, among others [23].

Although historically it was thought that baleen whales did not accumulate metals, recent data suggest they can. For example, North Atlantic right whales (*Eubalaena glacialis*) accumulate high levels of chromium [16]. Currently, the only data regarding metal concentrations in tissues of southern right whales come from dead calves [24,25]. Here we present results from a study in which we collected skin biopsies from live female southern right whales at Península Valdés and analyzed them for metals and selenium. To our knowledge this is the first set of data on metal levels in live individuals from this species and thus, our study serves as a valuable baseline.

## Materials and Methods

### Study area and biopsy sampling

**Figure 1:**
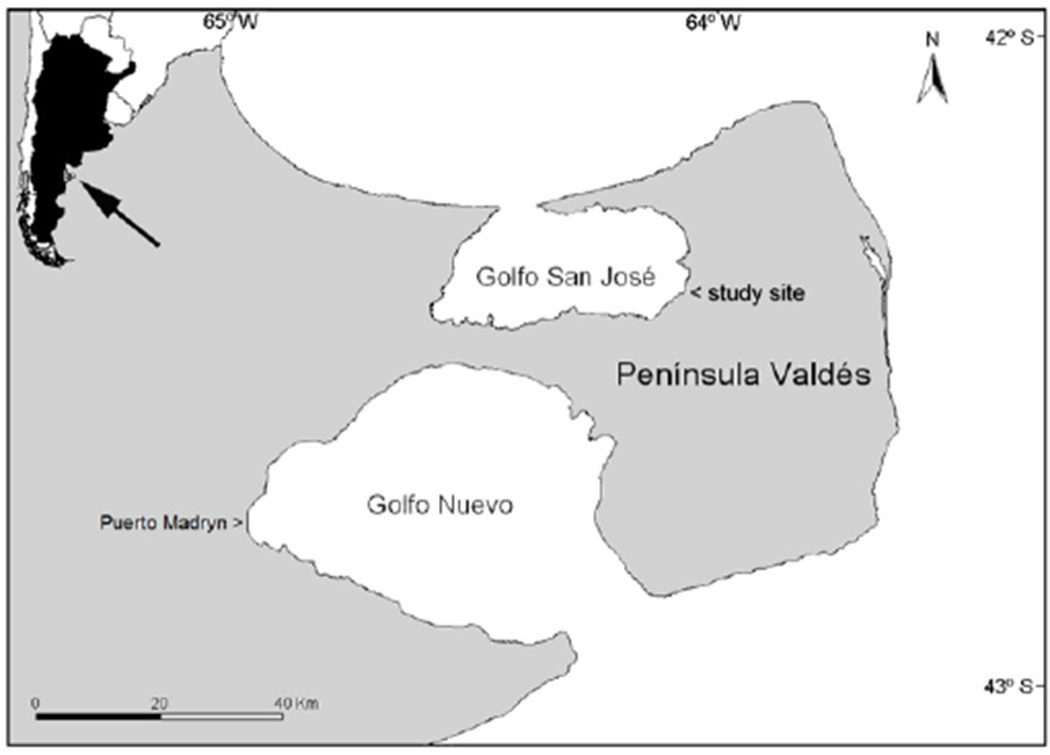
Map of Península Valdés, Argentina showing the San José Gulf, and the study site where the samples were collected, as well as the nearby city of Puerto Madryn, located on Golfo Nuevo. Inset shows the position of Península Valdés on the South American coast.

Biopsies were collected from ten adult female southern right whales in San José Gulf, Península Valdés, Argentina (42°30′; S, 64°00′; W; Figure 1), during the months of September and October of 2011. Adult females were recognized by the close proximity of a calf over an extended period of time. Animals were approached slowly in a small rubber boat. Prior to biopsying, the callosity patterns on the head of each whale were photographed to avoid resampling the same individual [26]. Whales were biopsied on their flanks using a crossbow and a dart, following standard methods described in Brown et al. [27]. A 60mm x 6mm stainless steel cylindrical biopsy dart was used. All tissue samples were immediately frozen after collection, stored in a liquid nitrogen dewar and shipped frozen to the laboratory.

### Metal analysis

Whale skin samples were analyzed for total levels of antimony (Sb), arsenic (As), barium (Ba), beryllium (Be), cadmium (Cd), chromium (Cr), cobalt (Co), copper (Cu), gold (Au), lead (Pb), lithium (Li), manganese (Mn), molybdenum (Mo), nickel (Ni), selenium (Se), silver (Ag), strontium (Sr), tin (Sn), titanium (Ti), uranium (U) and zinc (Zn) using a Perkin-Elmer/Sciex ETAN inductively coupled plasma-mass spectrometer (ICP-MS) according to EPA Method 6020A [28]. Aluminum (Al), iron (Fe), and magnesium (Mg) were measured using inductively coupled plasma-optical emission spectrometry (ICP-OES) according to EPA Method 6010B [29]. Mercury (Hg) was analyzed by thermal decomposition atomic absorbance using a Milestone DMA-80 according to EPA Method 7473 [30].

For analysis by ICP and ICP-MS, samples were rinsed with deionized water and allowed to air dry in a laminar flow hood to minimize contamination. Approximately 0.1 g of tissue was placed in a digestion vessel, 2 ml of Optima grade nitric acid (Fisher Scientific, Pittsburg, PA) was added, the vessel placed in a hot block, and refluxed at 95°C for 4 hours. The sample was cooled, 2 ml Optima grade hydrogen peroxide (Fisher Scientific, Pittsburg, PA) and deionized water (3:2 v/v) was added, heated until the effervescence subsided, cooled, and brought up to a final volume of 20 ml.

Standard quality assurance procedures were employed (Table 1) and include the analysis of standard reference materials, a duplicate sample and a pre-digestion spike. Instrument response was evaluated initially and after the 10 samples, using commercially available calibration verification standards (Claritas PPT multi-element solution 2 - Spex CertiPrep, Metuchen, NJ; individual elemental standards-SCP Science, Baie-D’Urfe, Quebec, Canada) and a blank. All calibration verifications (n=5) were within the acceptance criterion of 85-115 % recovery and the all preparation blank values were below 3x the limit of detection. Standard reference materials (DOET-4 and DORM-3-NIST, Charleston, SC) were used to assess method performance, where applicable. Interference check solutions (ICS A and ICS A+B- High Purity Standards, Charleston, SC) were analyzed with all sample runs to check for matrix effects which might be interfering with sample analysis.

The mean limit of detection (LOD) and project quantitation limit (PQL) values are presented in Table 1, since there is slight variability between samples due to differences in sample mass. The LOD was the lowest analyte concentration likely to be reliably distinguished from the blank and at which detection is feasible. The LOD was previously determined by utilizing both the measured blank and test replicates of a matrix matched sample known to contain a low concentration of analyte. Additionally, a series of PQL samples was run for each element to assess low level analytical performance, with an acceptance criterion of 50-150%. All samples were diluted 2x for analysis by ICP-MS.

Tissue concentrations are reported in μg/g per wet weight of tissue (μg/g ww). Some concentrations in the literature are reported as per dry weight of tissue and do not include information on water content. Hence, in order to roughly compare our data with those studies, we transformed literature values to per wet weight values by multiplying them by a factor of 0.25, which corresponds to a typical dry/wet weight ratio of most tissues [31].

## Results

Average tissue concentrations (μg/g ww) for each element are summarized in Tables 2 and 3 and are expressed as the mean ± standard deviation of the mean. The minimum and maximum values measured are also indicated. When calculating the mean of each element, values that were reported as non-detectable were replaced by ½ the limit of detection value for that given element. Among nonessential metals, A1 had the highest value with an average of 9.75 ± 2.7 μg/g tissue (w/w). U and Co were not detected in any of the samples. The remaining nonessential metals measured (Au, Sn, Cd, Li, Sb, Ag, Be, Hg, As, Pb, Ni, Ba, Cr, Sr and Ti) had averages that ranged from 0.11 to 3.95 μg/g tissue (w/w). Nonessential metal values in decreasing order of concentration are: Al>Ti>Sr>Cr>Ba>Ni>Pb >As>Au>Sn>Cd, Li, Sb>Ag>Be, Hg. Within the essential metals/element (i.e. selenium), Mg had the highest value with an average of 187.02 ± 9.1 μg/g tissue (w/w). Average tissue levels of Mo, Mn, Cu, Se, Fe and Zn ranged from 0.02 to 14.71 μg/g tissue (w/w).

## Discussion

Our study is the first to report metal concentrations in southern right whale skin from live adult animals. Overall, metal concentrations were generally low. Two previous studies investigated metals in southern right whales calves found dead on the beach in PV. One study reported metals in liver, kidney and muscle of a single calf [24]. The second study reported liver and kidney levels in 45 dead calves [25]. Table 4 shows a comparison between our findings and those of the previous studies. Overall, we found Al, Cd, Hg, Ni and Pb concentrations similar to those in the dead calves, but lower Cu, Fe, Mn and Zn concentrations.

These lower concentrations could be due to differences in the specific tissues evaluated. We measured skin, while previous studies focused on liver, kidney and muscle. Metals are known to preferentially accumulate in these internal organs more than the skin [32]. In minke whales (*Balaenoptera acutorostrata*), Cr and Cd skin levels correlate with those found in the liver [33] suggesting that skin levels could potentially reflect levels of internal organs. However, in a study in bowhead whales (*Balaena mysticetus*) [34] that compared concentrations of essential and nonessential elements in skin biopsies versus muscle, liver, blubber and kidney, skin values were not able to predict or correlate with the values measured in internal organs. In the case of southern right whales, there are no data available on metal concentrations in internal organs from adult whales to determine whether such a correlation exists or not. More data is needed to answer this important toxicological question.

Aluminum (Al) levels were of particular interest because of the presence of an Al smelter in the nearby city of Puerto Madryn, located on Golfo Nuevo, the southern Gulf of PV (Figure 1). Nine out of ten animals had detectable levels of Al. The levels found were comparable to those measured in liver and kidney of dead calves from PV [25]. Lower or similar levels of Al to those measured in our study were also found in liver, kidney and brain of juvenile gray whales (*Eschrichtius robustus*) from a subsistence harvest [35]. However, they were higher than those reported in livers of bowhead whales [36]. Taking into account that these studies measured Al in different tissues and hence direct comparisons are not possible, the Al concentrations we found appear to be low. These data would suggest that southern right whales are not experiencing higher exposure to Al as a result of the nearby smelter.

Previously we investigated Cr levels in skin biopsies from North Atlantic right whales [16], a species closely related to southern right whales. The average Cr levels in North Atlantic right whales were approximately ten times higher (7.1 μg/g ww) than those we found in southern right whales (0.64 μg/g ww). Similar protocols were used in each study. The explanation for this difference is uncertain, but is likely due to the fact that North Atlantic right whales live along one of the most industrialized coasts in the world while there is no significant chromium industry around PV.

Only a few studies have reported metal levels from skin biopsies of other baleen whales. De Luna and Rosales-Hoz [37] measured As, Fe, Mn, Pb, Se and Zn in skin of calf, juvenile and adult gray whales at Ojo de Liebre Lagoon, Mexico. Levels of As were similar to those in our study but the Pb level they report for adult gray whales is much higher than our study (~3.75 μg/g ww vs. 0.15 μg/g ww). This could be explained by differences in feeding behavior between gray and southern right whales. Gray whales are benthic bottom feeders that filter sediments in order to obtain their prey [38]. Higher levels of Pb could be due to a higher exposure resulting from the ingestion of sediments, which contain metals from natural or anthropogenic sources. Fe, Mn, Se and Zn levels in our study were higher than the levels found in these gray whales. This difference could be due to a low sample number (n=3) in the De Luna and Rosales-Hoz [37] study but could also reflect differences among sampling sites, ingested prey or species-specific differences in the toxicokinetics of these elements.

Ba, Cu, Cd, Cr, Mn, Hg, Sr, Se and Zn were measured in skin of 39 female minke whales in the southern hemisphere [33]. Our data are consistent with their Cu, Cr, Hg, Sr and Zn concentrations but different for Ba, Cd, Mn and Se. The biggest difference is with Ba, which was almost ninety-fold higher in our study (0.004 μg/g ww vs. 0.35 μg/g ww). Barium sulfate is widely used to make drilling fluids used in oil and gas extractions [39], an activity that is very prevalent in the Patagonia region surrounding PV. The higher levels of Ba in southern right whale might reflect a higher exposure to these Ba-containing compounds. However, barium sulfate is insoluble in water. An interesting, albeit untested, possibility is that right whales could be ingesting undissolved barium particles from the water column. Studies in rats suggest that once in the digestive tract, insoluble barium compounds could be slowly absorbed [40]. Mn was thirteen times higher in our study (0.02 μg/g ww vs. 0.27 μg/g ww) and Se was almost four-fold higher in their study (7.75 μg/g ww vs. 2.06 μg/g ww). Mn and Se are essential elements and thus, their absorption and excretion are tightly regulated. As mentioned earlier, these differences could be attributed to species-specific differences. Even though Cd was ten times higher in our study (0.004 μg/g ww vs. 0.04 μg/g ww) these values are still considered low.

In summary, this is the first study to report metal concentrations in live southern right whales. Overall, our data suggest that metal concentrations in southern right whales from PV are low and thus, could provide a valuable baseline for metals in skin tissue of this species. These low concentrations should not necessarily be interpreted as being safe since the effects of metals in marine mammals are largely unknown. Future work is aimed at providing more data from live southern right whales, including females, males as well as other age classes and reproductive status.

## Figures and Tables

**Table 1:** Quality assurance and quality control data for element analysis.

Element	LOD^a^	PQL^b^	Calibration Blank^c^ n=5	Duplicate (RPD) (%) n=1	LCS Recovery (%) n=1	Spike Recovery (%) n=1	SRM^d^ Recovery (%) DORM-3/DOLT-4 n=1

Aluminum (Al)	2.3	18.0	ND^e^	3.6	95.1	98.3	87.3
Antimony (Sb)	0.05	0.18	ND	BDL	103.4	109.9	N/A
Arsenic (As)	0.09	0.18	ND	BDL	97.9	107.6	99.8
Barium (Ba)	0.09	0.18	ND	5.9	98.1	104.9	N/A
Beryllium (Be)	0.02	0.19	ND	BDL	108.3	105.6	N/A
Cadmium (Cd)	0.02	0.18	ND	BDL	94.4	101.3	102.5
Chromium (Cr)	0.18	0.46	ND	8.9	107.8	94.7	116.7
Cobalt (Co)	0.02	0.18	ND	BDL	97.1	98.8	N/A
Copper (Cu)	0.05	0.18	ND	13.8	93.7	97.4	86.6
Gold (Au)	0.09	0.18	ND	BDL	95.9	96.8	N/A
Iron (Fe)	2.30	18.0	ND	1.5	92.0	95.6	92.5
Lead (Pb)	0.05	0.18	ND	4.2	93.3	97.3	107.9
Lithium (Li)	0.05	0.18	ND	BDL	106.2	106.1	N/A
Magnesium (Mg)	2.30	18.0	ND	9.6	96.4	97.4	N/A
Manganese (Mn)	0.02	0.18	ND	18.7	104.6	106.0	80.9
Mercury (Hg)	0.02	0.02	ND	99.4	96.7	105.1	98.8
Molybdenum (Mo)	0.02	0.18	ND	BDL	105.4	108.5	N/A
Nickel (Ni)	0.05	0.18	ND	19.6	95.2	95.8	88.3
Selenium (Se)	0.05	0.18	ND	1.9	101.4	104.4	103.2
Silver (Ag)	0.02	0.18	ND	BDL	107.2	110.7	92.9
Strontium (Sr)	0.02	0.18	ND	1.5	104.0	112.4	N/A
Tin (Sn)	0.05	0.18	ND	13.6	110.0	115.0	N/A
Titanium (Ti)	0.05	0.18	ND	17.9	112.1	110.3	N/A
Uranium (U)	0.02	0.18	ND	BDL	103.1	105.9	N/A
Zinc (Zn)	0.18	0.92	ND	13.9	97.2	107.7	98.2

a LOD = limit of detection (ppm, mean)

b PQL = project quantitation limit (ppm, mean)

c Calibration blank: ppm, mean

d SRM = standard reference material

e ND = not detected

**Table 2:** Concentrations (μg/g ww) of nonessential metals in skin biopsies from ten adult female southern right whales. Data represent the mean ± standard deviation of the mean and the range of minimum-maximum values measured.

Element	Mean Range	n (nd)^a^	Detection Limit^b^	Element	Mean Range	n (nd)^a^	Detection Limit^b^

**Al**	9.75 ± 2.7 3.1-24.8	10 (9)	2.3	**Sn**	0.07 ± 0 0.06-0.18	10 (7)	0.05
**Ti**	3.95 ± 0.2 3.5-5.72	10 (10)	0.05	**Cd**	0.04 ± 0 0.03-0.21	10 (6)	0.02
**Sr**	0.87 ± 0.1 0.54-1.46	10 (10)	0.02	**Li**	0.04 ± 0 0.05-0.08	10 (3)	0.05
**Cr**	0.64 ± 0.2 0.19-2.15	10 (9)	0.18	**Sb**	0.04 ± 0 0.07-0.08	10 (2)	0.05
**Ba**	0.32 ± 0.1 0.1-1.08	10 (9)	0.09	**Ag**	0.02 ± 0 0.06	10 (1)	0.02
**Ni**	0.19 ± 0 0.06-0.51	10 (10)	0.05	**Hg**	0.01 ± 0 0.02-0.03	10 (2)	0.02
**Pb**	0.15 ± 0 0.08-0.53	10 (8)	0.05	**Be**	0.0.1 ± 0 0.03	10 (1)	0.02
**As**	0.11 ± 0 0.19-0.41	10 (3)	0.09	**U**	ND^c^	10 (0)	0.02
**Au**	0.09 ± 0 0.23-0.33	10 (2)	0.09	**Co**	ND^c^	10 (0)	0.02

a n (nd) = Total number of samples (Number of samples with detectable levels)

b Detection limit = μg/g

c ND = Not detected

**Table 3:** Concentrations (μg/g ww) of essential metals and one essential element (Se) in skin biopsies from ten adult female southern right whales. Data represent the mean ± standard deviation of the mean and the range of minimum-maximum values measured.

Element	Mean Range	n (nd)^a^	Detection Limit^b^	Element	Mean Range	n (nd)^a^	Detection Limit^b^

**Mg**	187.02 ± 9.1 138.1-230.8	10 (10)	2.3	**Cu**	0.35 ± 0.1 0.09-0.67	10 (10)	0.05
**Zn**	14.71 ± 0.6 12.79-19.67	10 (10)	0.18	**Mn**	0.27 ± 0.1 0.1-0.74	10 (10)	0.02
**Fe**	7.24 ± 3 2.3-32.3	10 (7)	2.3	**Mo**	0.02 ± 0 0.02-0.06	10 (6)	0.02
**Se**	2.06 ± 0.4 0.7-4.4	10 (10)	0.05				

a n (nd) = Total number of samples (Number of samples with detectable levels)

b Detection limit = μg/g

**Table 4:** Summary of reported metal concentrations (μg/g ww) in tissues of southern right whales. These concentrations reflect the minimum and maximum values measured in each study.

Studies	This Study n=10 Adult Females	Gil et al. (2006) n=1 Calf			Rosas et al. (2012) n=45 Calves	

Metal/Organ	Skin	Liver	Kidney	Muscle	Liver	Kidney

**Al**	3.1-24.8	NM^b^	NM^b^	NM^b^	1.0-26.5	1.1-22.7
**Cd**	0.03-0.21	0.04	0.04	0.04	ND^a^	ND^a^
**Cu**	0.09-0.67	18.6	5.7	2.6	1.88-264.41	1.67-5.65
**Fe**	2.3-32.3	NM^b^	NM^b^	NM^b^	25.5-184.48	16.1-120.6
**Hg**	0.02-0.03	ND^a^	0.04	0.04	NM^b^	NM^b^
**Mn**	0.1-0.74	NM^b^	NM^b^	NM^b^	0.09-2.9	0.15-3.33
**Ni**	0.06-0.51	NM^b^	NM^b^	NM^b^	0.13-0.22	0.1-0.94
**Pb**	0.08-0.53	0.13	0.12	0.13	ND^a^	ND^a^
**Zn**	12.79-19.67	NM^b^	54	83	0.23-303.01	11.42-45.67

a ND = not detected

b NM = not measured
